# Intelligent Fault Diagnosis of Hydraulic Multi-Way Valve Using the Improved SECNN-GRU Method with mRMR Feature Selection

**DOI:** 10.3390/s23239371

**Published:** 2023-11-23

**Authors:** Hanlin Guan, Ren Yan, Hesheng Tang, Jiawei Xiang

**Affiliations:** The College of Mechanical and Electrical Engineering, Wenzhou University, Wenzhou 325035, China; 21461440019@stu.wzu.edu.cn (H.G.); tanghesheng@wzu.edu.cn (H.T.); jwxiang@wzu.edu.cn (J.X.)

**Keywords:** hydraulic multi-way valve, intelligent fault diagnosis, maximum relevance minimum redundancy (mRMR), squeeze-excitation convolution neural network and gated recurrent unit (SECNN-GRU)

## Abstract

Hydraulic multi-way valves as core components are widely applied in engineering machinery, mining machinery, and metallurgical industries. Due to the harsh working environment, faults in hydraulic multi-way valves are prone to occur, and the faults that occur are hidden. Moreover, hydraulic multi-way valves are expensive, and multiple experiments are difficult to replicate to obtain true fault data. Therefore, it is not easy to achieve fault diagnosis of hydraulic multi-way valves. To address this problem, an effective intelligent fault diagnosis method is proposed using an improved Squeeze-Excitation Convolution Neural Network and Gated Recurrent Unit (SECNN-GRU). The effectiveness of the method is verified by designing a simulation model for a hydraulic multi-way valve to generate fault data, as well as the actual data obtained by establishing an experimental platform for a directional valve. In this method, shallow statistical features are first extracted from data containing fault information, and then fault features with high correlation with fault types are selected using the Maximum Relevance Minimum Redundancy algorithm (mRMR). Next, spatial dimension features are extracted through CNN. By adding the Squeeze-Excitation Block, different weights are assigned to features to obtain weighted feature vectors. Finally, the time-dimension features of the weighted feature vectors are extracted and fused through GRU, and the fused features are classified using a classifier. The fault data obtained from the simulation model verifies that the average diagnostic accuracy of this method can reach 98.94%. The average accuracy of this method can reach 92.10% (A1 sensor as an example) through experimental data validation of the directional valve. Compared with other intelligent diagnostic algorithms, the proposed method has better stationarity and higher diagnostic accuracy, providing a feasible solution for fault diagnosis of the hydraulic multi-way valve.

## 1. Introduction

A hydraulic multi-way valve refers to a multi-way combination valve composed of two or more directional valves as the main body and auxiliary devices, such as safety valves, one-way valves, and makeup valves, according to different working requirements. As the core component, hydraulic multi-way valves are widely applied in industries such as engineering machinery, mining machinery, metallurgy, and shipbuilding. Due to the harsh working conditions, including dust, high temperatures, vibrations [1], and prolonged operation, hydraulic multi-way valves are prone to malfunctions. Moreover, the hydraulic system it operates in is very complex, so it is difficult to determine the type or degree of fault of the hydraulic multi-way valve based on engineering fault appearances [2]. Consequently, the likelihood of malfunctions has increased. When a fault occurs, it becomes challenging to quickly determine the cause and accurately identify its location within the components. The internal performance will be damaged by this delay in diagnosis, and eventually, the hydraulic equipment fault will be aggravated. As a result, economic losses and safety issues are generated, and even staff members may be harmed, including injury or death [3]. Therefore, it is necessary to conduct research on fault diagnosis of hydraulic components, especially hydraulic multi-way valves.

There are three main methods for diagnosing faults in hydraulic components: model-based, signal processing, and artificial intelligence algorithms [4]. Model-based diagnostic methods require accurate system models to be known. It is generally divided into the parameter estimation method [5], the state prediction method [6], and the parity space method [7]. For hydraulic systems, the basic idea is to combine theoretical modeling and parameter identification and determine faults based on the deviation between parameter estimates and normal values. For example, Liu et al. established a mathematical model based on the dynamic equation of a hydraulic servo system. According to the changes in the model parameters, the fault mode and location of the hydraulic servo system can be diagnosed and isolated, and its effectiveness can be verified through experiments [8]. Similarly, Samadani et al. established a nonlinear mathematical model of a servo electro-hydraulic system, estimated the original values of fault parameters through recurrence quantification analysis, and identified the severity of system faults [9]. Model-based fault diagnosis methods are suitable for systems with known, precise mathematical models, but it is very difficult to establish an accurate mathematical model of the system under actual operating conditions. Taking hydraulic components as an example, Reynolds number, laminar or turbulent flow, channel geometry, flow coefficient, etc., are time-varying and susceptible to temperature, interference, and noise, resulting in complex mapping relationships between faults and model parameters [10]. It is usually necessary to combine it with other methods to improve the performance of fault diagnosis systems.

The raw detection signals collected generally not only have a large amount of data, but are also sensitive to the operating environment, so it is necessary to further process the collected detection signals. The diagnostic method of signal processing mainly uses some mathematical transformations to extract fault features from the original signal. For example, Goharrizi et al. used the discrete wavelet transform to refine the decomposed pressure signal and establish a feature model that can effectively detect internal leakage and its severity [11]. The author also decomposed the pressure signal on one side of the valve-controlled hydraulic actuator into intrinsic mode functions (IMFs) and performed the Hilbert transform on each IMF to obtain the instantaneous amplitude. By comparing the root mean square value of the instantaneous amplitude related to the IMF with the root mean square value under normal working conditions, a feature mode for detecting internal leakage and its severity was established [12]. However, due to the complexity of hydraulic systems or components themselves, the variability of operating conditions, and the harsh working environment, a large amount of fault information is hidden in the original signals. Simple signal processing methods make it difficult to effectively highlight or amplify fault feature information.

With the development of sensors and computing systems, the amount of data describing device status information, including fault information, has grown exponentially. A large number of artificial intelligence data-driven fault diagnosis methods have emerged [13,14,15,16,17,18]. In traditional artificial intelligence algorithms, such as expert systems [19], fuzzy diagnosis [20], and neural networks [21,22,23], corresponding expert knowledge or a large number of fault data samples are necessary. However, for most complex equipment systems, including hydraulic systems, it is difficult to obtain a sufficient number of regular fault data samples without missing outliers due to limitations in the working environment, traditional technology, and other conditions. Therefore, research on deep learning has been carried out. Taking convolutional neural networks (CNN) as an example, they have been commonly used in fault diagnosis of hydraulic systems or components instead of using pressure sensors or flow sensors to measure some values to reflect the hydraulic system failure. For example, Huang et al. used CNN to extract fault features from fault signal samples of the hydraulic system to realize fault diagnosis of the hydraulic system and verified the effectiveness of the method through experiments [24]. Tang et al. took the fault of a hydraulic plunger pump as an example, improved CNN to extract and identify fault features, and obtained results with high diagnostic accuracy [25]. Although the aforementioned methods can improve diagnosis performance to some extent, the time series relationships of data are disregarded. The collected data is a time series and contains abundant time-dependent properties, which can provide additional useful information to distinguish between different fault patterns.

CNN is a hierarchical network structure and cannot model changes in time series. The time order of the data in the sample is very important for fault identification, so Recurrent Neural Networks (RNN) have been widely used in time series information processing. Although RNN is suitable for processing sequence data, it is affected by short-term memory, so gate structures are introduced to selectively store information to reduce short-term memory effects, such as the Long Short-term Memory Network (LSTM) and Gated Recurrent Unit (GRU). GRU alleviates the problem of gradient disappearance in RNN. It combines the forgotten gate and the input gate in LSTM into an update gate, which makes it simpler, has fewer parameters, and improves training efficiency.

By combining the CNN and RNN algorithms, the time-space information of data can be obtained, and multi-dimensional mining of fault features in a signal can be realized. Liu et al. designed a 1DCNN-GRU that combines the advantages of the spatial processing capabilities of CNN and the time series processing capabilities of GRU. The effective features are extracted by CNN adaptive, and the GRU further learns the features processed by CNN. The results show that the method can adaptively extract spatial and time-dependent features from the original vibration signals, and the diagnosis accuracy is higher than 99% [26]. Similarly, Liao et al. proposed a fault diagnosis system for hydroelectric generating units based on 1DCNN-GRU. The performance of the proposed method is verified by comparing it with the results of other machine learning methods. Moreover, the fault diagnosis method has been successfully applied to engineering practice after being trained with actual vibration signals [27]. Although the methods mentioned effectively improve the performance of fault diagnosis, the weight relationship among fault features are neglected.

In order to avoid repetition and full convolution, resources are reassigned, i.e., weights, based on the importance of the attention object to highlight certain important features [28,29]. In CNN, attention mechanisms can be divided into two types: channel attention and spatial attention. Channel attention determines the weight relationship between different channels, with the weight of key channels being increased and the weight of less effective channels being suppressed; spatial attention is the determination of the weight relationship between different pixels in the spatial neighborhood. The weight of key area pixels is increased, while the weight of unnecessary areas is reduced. Hu et al. proposed a Squeeze-and-Excitation (SE) module that significantly improves the ability of deep learning methods to learn image features [30]. Tang et al. proposed a deep neural network fault diagnosis method with an attention mechanism. The SE module and deep neural network are combined to realize the intelligent diagnosis of adaptive feature extraction of rolling bearings. The results show that the method can achieve a 100% fault recognition rate under variable speed conditions and has strong noise resistance, adaptability, and robustness [31]. Compared with CNN and its variants, CNN with a SE module can improve the fault diagnosis accuracy of the model, while the parameters and the computation in this structure are comparatively less. It may provide an effective reference for fault diagnosis of hydraulic components.

The fault feature information contained in signals obtained on hydraulic components, especially vibration signals, is often masked by noise information, but noise and fault information usually have different frequency distribution characteristics. Similar to filters, in convolutional operations, convolutional kernels of different feature channels can be used to extract feature information from different frequency bands in vibration signals. If the weights of each feature channel after adjusting the convolution operation can be targeted and given higher weights to feature channels that are beneficial for classification, but lower weights to feature channels that are not beneficial for classification, then the final extracted features will better reflect fault information. Therefore, a one-dimensional CNN fault diagnosis model based on feature channel weight adjustment is designed. On the basis of one-dimensional convolutional networks, the SE module is introduced to adjust the weight of feature channels, so SECNN is obtained. Combining SECNN and GRU, an intelligent fault diagnosis method for the hydraulic multi-way valve is proposed. By obtaining more global and multi-dimensional information, the robustness of feature extraction to changes in operating conditions is enhanced, and the generalization ability of the model under different operating conditions is improved.

The main contributions of this paper are as follows:

1. The simulation model of the hydraulic multi-way valve is established, and the fault data of the multi-way valve is obtained through simulation as an extension of the experimental data.
2. Based on the shallow statistical features, the spatial and temporal multi-dimensional fault features are extracted, and the fault features are weighted adaptively combined with the attention mechanism.
3. A fault diagnosis method for hydraulic multi-way valves based on CNN, SE, and GRU is proposed, which effectively realizes fault diagnosis for hydraulic multi-way valves even under variable operating conditions.

This paper consists of five parts, including the introduction. Section 2 introduces the fault diagnosis method of the hydraulic multi-way valve. In Section 3, the simulation model and the experimental platform are designed, and fault data are obtained. Section 4 analyzes the effectiveness of the proposed method; Section 5 concludes this study.

## 2. Methods

**Figure 1:**
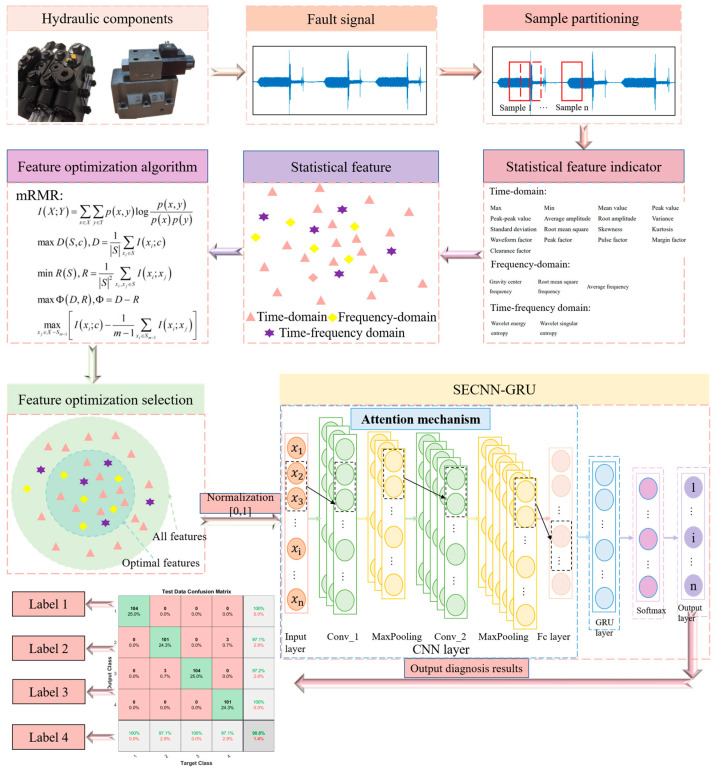
Hydraulic valve fault diagnosis process.

An effective intelligent fault diagnosis method is proposed using an improved Squeeze-Excitation Convolution Neural Network and Gated Recurrent Unit (SECNN-GRU). This method includes: fault signal acquisition, sample partitioning, statistical feature indicator, statistical feature, feature optimization algorithm, feature optimization selection, and SECNN-GRU, as shown in Figure 1. Firstly, the window sliding is used to enhance the fault sample data of the fault signal, and then the shallow statistical features are extracted from the data containing fault information. Following the fault feature selection steps, fault features with a high correlation with fault types are selected using the mRMR algorithm. Subsequently, the convolution operation is carried out on the optimized fault features, and the weighted spatial dimension features are obtained by adding SE blocks, which are then input into the GRU network for timing feature processing. The fault characteristics of hydraulic multi-way valves are extracted in the time dimension. Finally, the final fault diagnosis results are obtained by a classifier.

The steps involved in the proposed approach are as follows:

(1) Using a data acquisition system, the healthy signal and fault signals of the hydraulic valve under different operating conditions are obtained.
(2) The window sliding is used to enhance the fault sample data of the fault signal, and then the shallow statistical features (i.e., time-domain, frequency-domain, and time-frequency domain features) are extracted from the data containing fault information.
(3) Conduct an mRMR feature selection algorithm analysis on the shallow statistical features of the hydraulic valve obtained under different operating conditions to assess the correlation with faults. This analysis identifies the optimal features among these features for the subsequent processing.
(4) In this step, the CNN is carried out on the optimized fault features, and the weighted spatial dimension features are obtained by adding an SE block, which is then input into the GRU network for timing feature processing. The fault features of hydraulic multi-way valves are extracted in the time dimension. Consequently, the final fault diagnosis results are obtained.

### 2.1. Fault Sample Data Enhancement

To obtain enough samples, the time window slip technique is used to segment the original data and construct the dataset. In order to prevent the loss of sample feature information caused by segmentation, the correlation of adjacent sequence signals is preserved to the maximum, and the samples are overlapped when the window slips. The total length of the pressure signal is L, the unit sample length is l, and the window shift length is s. Then, the number of samples n that can be divided by the current signal length is shown in Equation (1).

(1) $$n=\left[\frac{L-l}{s}+1\right]$$

where $\left[\cdot \right]$ represents a downward integral function.

### 2.2. Data Feature Extraction

The collected data usually uses feature extraction techniques such as time domain, frequency domain, or wavelet domain to extract fault information. In the proposed method, the pressure, flow, or vibration signals containing fault information collected for hydraulic multi-way valves in construction machinery make it difficult to establish direct mapping relationships with fault types. Therefore, it is necessary to first extract fault features. The time-domain, frequency-domain, and time-frequency domain feature indicators are used to extract the features of fault signals, as shown in Table 1. The table lists 17 time-domain, 3 frequency-domain, and 2 time-frequency domain features as statistical features.

### 2.3. Max-Relevance and Min-Redundancy for Feature Selection

The extracted features are shallow statistical features, which will reduce classification accuracy and waste computational resources during the operation process. Therefore, feature selection is applied to select a simplified feature set with good classification performance. In the proposed method, the Max-Relevance and Min-Redundancy (mRMR) algorithm is adopted; in the original feature set, a set of features with the highest correlation to the final output but the lowest correlation between features is selected. Maximizing the relevance between features and classification to solve the optimal feature combination problem: To improve the redundancy between features, the correlation between features is minimized [32,33]. The basic theory of the mRMR method is summarized in the following.

For fault classification problems, mutual information is used to measure the similarity between variables. Suppose two random variables X and Y, and their probability and joint probability density functions are$\;p\left(x\right)$, $p\left(y\right)$ and $\;p\left(x,y\right)$, respectively, then the mutual information between them is defined as follows:

(2) $$I\left(X;Y\right)=\sum_{x\in X}\sum_{y\in Y}p\left(x,y\right)\log\frac{p\left(x,y\right)}{p\left(x\right)p\left(y\right)}$$

Suppose $x_{i}$ and c represent single feature and class, respectively, the dimension of feature space S is $\left|S\right|$, and the mutual information between a single feature and class, then the maximum relevance criterion is expressed as:

(3) $$max\;D\left(S,c\right),D=\frac{1}{\left|S\right|}\sum_{x_{i}\in S}I\left(x_{i};c\right)$$

The minimum redundancy is used to select mutually exclusive features to remove the possible feature redundancy after the maximum relevance criterion. The mutual information between feature $x_{i}$ and $x_{j}$ is $\left(x_{i};x_{j}\right)$, then the minimum redundancy expression is:

(4) $$\min R\left(S\right),R=\frac{1}{{\left|S\right|}^{2}}\sum_{x_{i},x_{j}\in S}I\left(x_{i};x_{j}\right)$$

The above Equations (3) and (4) are integrated by addition and multiplication to optimize *D* and *R*, and the maximum relevance and minimum redundancy is obtained, which is expressed as follows:

(5) $$\max\emptyset \left(D,R\right),\emptyset =D-R$$

(6) $$\max\emptyset \left(D,R\right),\emptyset =D/R$$

Equation (5) can ensure that the relevance between features and categories reaches the maximum value and the relevance between features reaches the minimum value. Therefore, this formula condition is optimized. In practice, the incremental search method is used to find approximately optimal features. Assuming we already have a feature set $S_{m-1}$, the task is to find the m th feature from the remaining feature $X-S_{m-1}$, and maximize $\emptyset \left(\cdot \right)$ by selecting the feature. The increment algorithm optimizes the following expression:

(7) $$\underset{x_{j}\in X-S_{m-1}}{\max}\left[I\left(x_{j};c\right)-\frac{1}{m-1}\sum_{x_{i}\in S_{m-1}}I\left(x_{i};x_{j}\right)\right]$$

### 2.4. CNN Based on Attention Mechanism

CNN is mainly comprised of various layers, including the input layer, convolutional layer, pooling layer, fully connected layer, and output layer. The CNN leverages convolution kernels of varying sizes to extract deep spatial features from the raw data and reduce dimensionality. The convolution operation formula is:

(8) $$C=f\left(X*W+B\right)$$

where C represents feature output after convolution operation; $f\left(\cdot \right)$ represents activation function, and Relu is used in this paper. X represents the input data; ∗ represents the convolution operation; W is the weight of the convolution kernel; B for additive bias.

The pooling layer samples the features after the convolution operation and extracts the main features so as to reduce parameters and computation, and improve the robustness and running speed of the system. Maximum pooling is adopted in this paper, and its formula is as follows:

(9) $$P=f\left(\beta max\left(X\right)+B\right)$$

where P represents the output value after the pooling operation; β represents multiplicative bias; $\max\left(\cdot \right)$ represents the sampling function; X represents input data; B for additive bias.

The fully connected layer combines the extracted features, maps them to the space of sample markers, and finally realizes the output of classification results by combining them with the classifier. In this paper, the softmax classifier is adopted, and its formula is as follows:

(10) $$Y=softmax\left(X\right)=\frac{e^{X}}{\sum_{k=1}^{m}e^{X}}$$

where Y represents the classification value of the sample; X represents the node value of the neuron; m represents the total number of categories.

**Figure 2:**
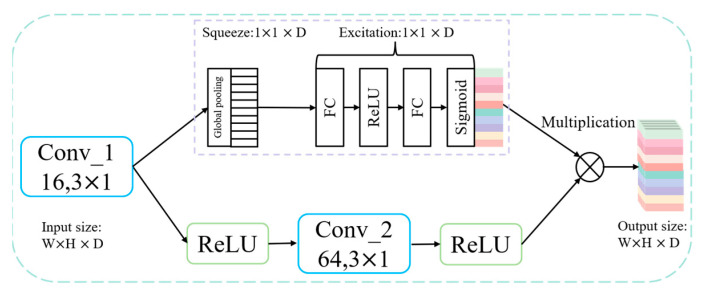
Schematic diagram of CNN combined with squeeze and excitation.

The CNN structure combined with the attention mechanism is shown in Figure 2, the weight of each feature channel is increased, and the Squeeze-Excitation (SE) Block is used to focus on the channel dimension. In order to accurately extract fault features, the weights of different channels are enhanced or suppressed: feature channels that are beneficial for classification are given higher weights, while feature channels that are not beneficial for classification are given lower weights.

There are three main steps in this process. First, the features of the spatial dimension are compressed to keep the number of channels unchanged. Global pooling is performed, and each two-dimensional feature channel is converted to a real number. Its mathematical expression is as follows:

(11) $$U_{k}=\frac{1}{H\times W}\sum_{i=1}^{H}\sum_{j=1}^{W}\mu_{ij}$$

Secondly, the excitation operation is carried out, which consists of two fully connected layers and Sigmoid function, and its mathematical expression is as follows:

(12) $$S=\sigma \left(W_{2}\delta \left(W_{1}U\right)\right)$$

where *S* is the output of the excitation operation, δ is the activation function Sigmoid, $W_{1}$ and $W_{2}$ are the two fully connected corresponding parameters, respectively, σ is the activation function Relu, and the dimension of the feature is first reduced and then increased.

Finally, the reweight operation is carried out to weigh the previous input features by channel to complete the redistribution of the original features on each channel.

### 2.5. A Brief Introduction to GRU

If the gradient disappears during the training process, the weights cannot be updated and the training fails. The gradient brought by the gradient explosion is too large, and the network parameters are greatly updated, and in extreme cases, the results will overflow. GRU alleviates the problem of gradient disappearance in RNN [34].

The current concealed state $h_{t}$ is linked to the concealed state $h_{t-1}$ in the previous moment and the current input $x_{t}$. The $h_{t-1}$ comprises the information at moment t−1 which is the historical information utilized while acquiring the present state. The resetting gate $r_{t}$ is employed to govern the transition from the historical information $h_{t-1}$ to the concealed potential state $\tilde{h_{t}}$ of the regression block at the present moment. $r_{t}\;$is designated as 0 or 1, signifying whether the entire historical information is utilized at the present moment. The higher the value of r, the greater the amount of information accessible from the previous moment. The mathematical expression is as follows:

(13) $$r_{t}=\sigma \left(W_{rx}x_{t}+W_{rh}h_{t-1}+b_{r}\right)$$

where $x_{t}$ represents the input at moment t, $W_{rx}$ and $W_{rh}$ represents the weight of $x_{t}$ and $h_{t-1}$, $b_{r}$ represents the bias of the resetting gate $r_{t}$, and $\sigma \left(\cdot \right)$ represents the Sigmoid function. The modeling process utilizes the update gate $z_{t}$ to regulate the extent of historical information incorporated. Analogous to the resetting gate $r_{t}$, a higher value of the update gate, leads to a greater utilization of past information within the loop block. The mathematical expression is as follows:

(14) $$z_{t}=\sigma \left(W_{zx}x_{t}+W_{zh}h_{t-1}+b_{z}\right)$$

where $W_{zx}$ and $W_{zh}$ represents the weight of $x_{t}$ and $h_{t-1}$, $b_{z}$ is the bias of the update gate $z_{t}$.

The specific calculation expression of the concealed potential $\tilde{h_{t}}$ is as follows:

(15) $$\tilde{h_{t}}=\tanh\left(W_{{\tilde{h}}_{t}}x_{t}+W_{{\tilde{h}}_{t}h}\left(r_{t}⊙h_{t-1}\right)+b_{h}\right)$$

where, $W_{{\tilde{h}}_{t}}$ and $W_{{\tilde{h}}_{t}h}$ represents the weight of $x_{t}$ and $h_{t-1}$, $b_{h}$ is the bias of the concealed potential $\tilde{h_{t}}$, $\tanh\left(\cdot \right)\;$is the tanh function, and ⊙ is element multiplication.

The specific calculation expression of the concealed $h_{t}$ at the present moment is as follows:

(16) $$h_{t}=\left(1-z_{t}\right)⊙h_{t-1}+z_{t}⊙{\tilde{h}}_{t}$$

## 3. Obtained Fault Data

### 3.1. Case1: Simulation and Experiment of the Hydraulic Multi-Way Valve

#### 3.1.1. Simulation Model

**Figure 3:**
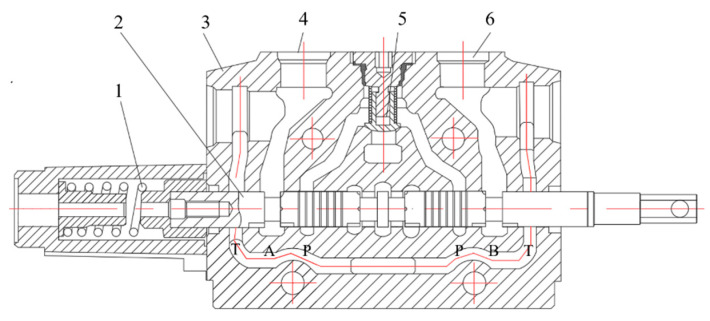
Hydraulic multi-way valve structure diagram; 1. Reset spring 2. Reversing valve spool 3. Valve body 4. Working oil port 5. Check valve 6. Working oil port.

The structure principle of the hydraulic multi-way valve applied in engineering machinery is shown in Figure 3. The hydraulic multi-way valve is an integrated valve composed of two or more directional valves, combined with safety valves, overload valves, makeup valves, diverter valves, brake valves, one-way valves, etc., used to control the movement of multiple actuator components. Here, A and B are the working oil ports, P is the oil inlet port, and T is the oil return port. The working principle of a hydraulic multi-way valve is based on the action of a piston, which drives the valve core to move in a specific direction, thereby changing the direction of hydraulic oil on the valve core to control the hydraulic system. When the spool moves to the right, P and T communicated with A and B, respectively; When the spool moves to the left, P and T are communicated with B and A, respectively. Usually, the working valve blades are configured in groups, and the overflow valve is built into the inlet valve block to achieve pressure compensation by bypassing the main oil circuit. As a logic component, when the multi-way valve stops working and each valve is in the middle position, the valve bypasses the main oil circuit with a specific compensation pressure. When the working conditions change, the bypass overflow valve will reduce the bypass flow under the load pressure to provide the required flow according to the load pressure.

**Figure 4:**
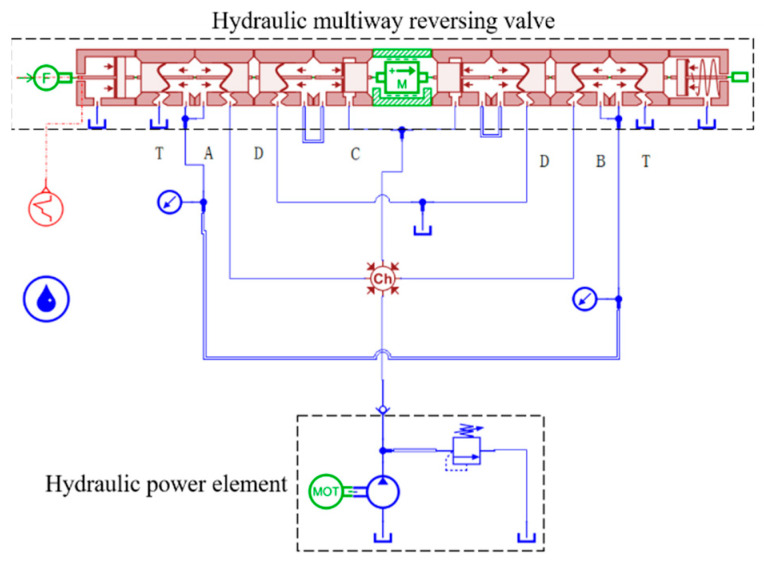
AMESim simulation model of a hydraulic multi-way valve.

According to the structural characteristics and working principle of the hydraulic multi-way valve, a simulation model of the hydraulic multi-way valve is built by using the sub-modules of the hydraulic library, hydraulic component design library, plane one-dimensional mechanical library, and signal library in AMESim software (https://plm.sw.siemens.com/en-US/simcenter/systems-simulation/amesim/), as shown in Figure 4. The simulation model includes hydraulic power components, hydraulic multi-way reversing valves, and hydraulic auxiliary components. The hydraulic pump provides the required oil pressure and flows for the entire hydraulic system, and the relief valve at the pump outlet is used to limit the maximum pressure of the system. The multi-way reversing valve considers the quality of the main spool element, different throttling forms, and some simplified forms of the model. The signal source applies driving instructions to the multi-way valve spool to realize the switching of the multi-way valve between different working oil channels. The simulation parameters are set as shown in Table 2.

**Figure 5:**
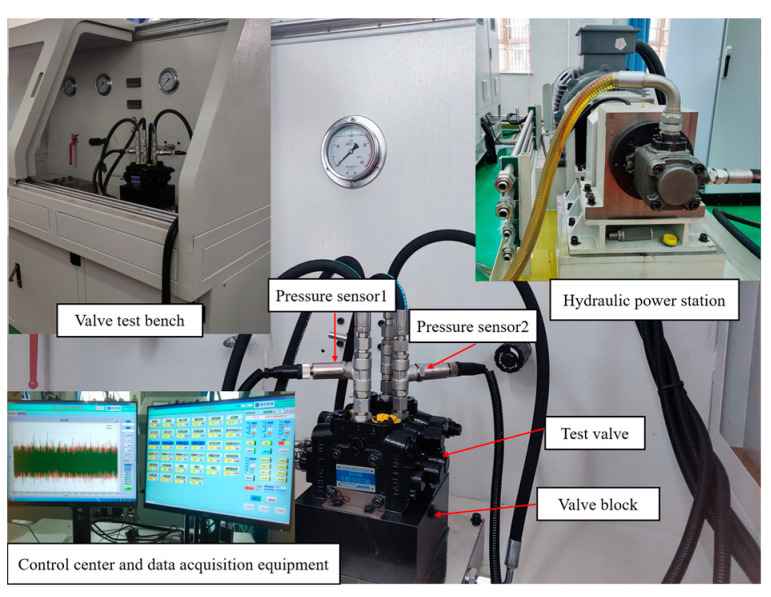
Test rig used for the experiments.

Combined with the actual engineering conditions, the parameters of the simulation model were analyzed and adjusted, and the pressure characteristics were tested through the experiment of the hydraulic multi-way valve, as shown in Figure 5.

The pressure for this study was set at 3.5 MPa, the sampling time was 30 s, and the sampling frequency was 6 kHz. The pressure sensors (model KS-E-Z-B06C-M-V-530, GEFRAN, Singapore) were installed at the inlet port A and the return port T of the hydraulic multi-way valve.

**Figure 6:**
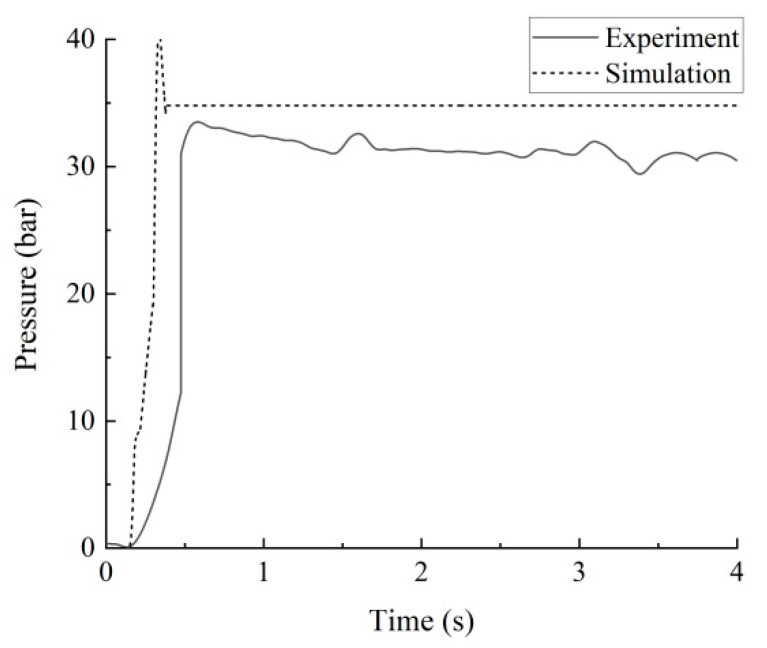
Experimental and simulation comparison diagrams of hydraulic multi-way valve pressure.

The comparison curve of pressure characteristics between simulation and experiment is shown in Figure 6, where the horizontal coordinate represents a reversing action (4 s) of the multi-way valve, and the vertical coordinate represents the oil pressure at the inlet port A. It can be seen that the trend of simulation data and experimental data is highly consistent. Due to some unavoidable interference in the experiment, the pressure data have certain deviations and fluctuations in the oil pressure setting value attachment. On the whole, the two data have a high correlation, which shows that the simulation model mapped the actual pressure characteristics of the hydraulic multi-way valve.

#### 3.1.2. Fault Models

Hydraulic multi-way valves, as important hydraulic components, may experience problems such as oil leakage and unstable pressure during operation. In this paper, three fault modes and a normal state of hydraulic multi-way valves are defined, as shown in Table 3.

In the AMESim simulation model, different fault injection methods are used to simulate the corresponding fault conditions. The air content in the oil was 1% (normal 0.1%), 5 N/cm (normal 10 N/cm), and 0 N/cm to simulate cavitation failure, moderate spring failure, and severe spring failure, respectively.

#### 3.1.3. Simulated Fault Data

**Figure 7:**
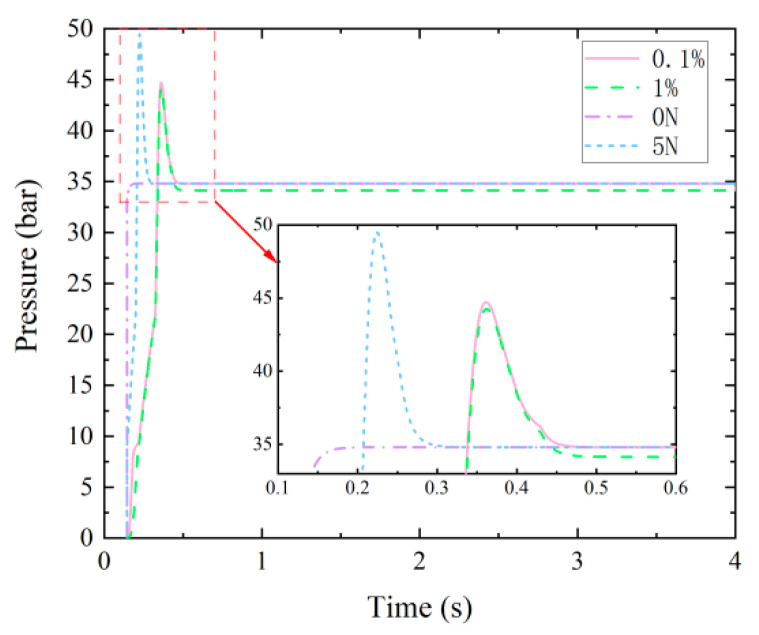
Pressure signals of hydraulic multi-way valves under different fault states.

Due to the small number of experimental fault samples, the data obtained from the simulation model is supplemented. In the simulation model, the pressure signal of working oil port A is used as the data source for fault diagnosis. The simulation time is set to 4 s, the data collection frequency is set to 6 kHz, and the fault sample space size is 1 × 24,000 × 1. The pressure signals under different fault modes were compared, as shown in Figure 7. The pressure signals are similar under different fault states, making it difficult to diagnose faults using traditional methods.

### 3.2. Case2: Experiment of the Hydraulic Directional Valve

#### 3.2.1. Working Principle

**Figure 8:**
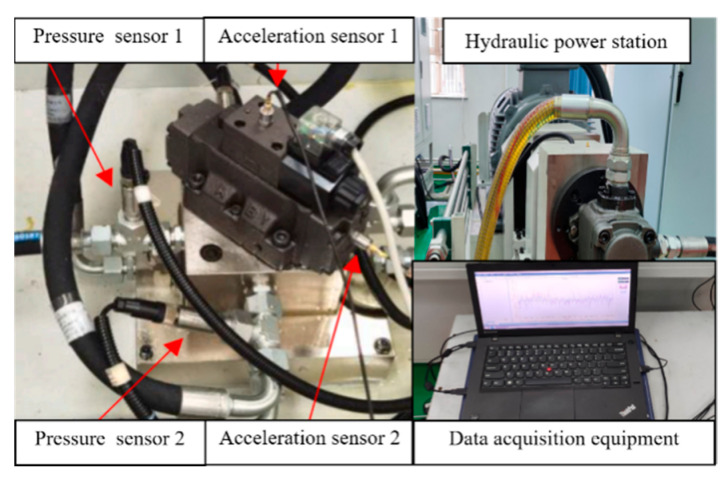
Experimental platform for a hydraulic directional valve.

The essence of a hydraulic multi-way valve is a hydraulic directional valve, so the effectiveness of the proposed method is further verified by the experimental data of a common directional valve. The working principle of the hydraulic directional valve is to use the relative operation between the valve core and the valve body to change the direction of the oil connection, so that the actuator moves, stops, or reverses. The test platform is shown in Figure 8. Different types and severity of reversing valve faults were set up in the experiment. The hydraulic valve reversing interval was 4 s, and the cycle was 8 s. The pressure for this study was set at 2 MPa, the sampling time was 30 min, and the sampling frequency of the acceleration and pressure sensor was 6 kHz. The pressure sensors (model KS-E-E-Z-B06C-M-V-530, GEFRAN) were installed at the working ports A and B of the directional valve, respectively. The acceleration sensors (model HD-YD-185, ECON, Singapore) were installed on the top and side of the directional valve.

#### 3.2.2. Fault Models

In order to verify the effectiveness of the proposed method, 6 types of faults of the hydraulic directional valve were designed, as shown in Table 4. The failure of the hydraulic directional valve mainly includes the wear of the spool of different severity and the fatigue failure of the return spring. In the experiment, the failure of the hydraulic directional valve was artificially manufactured, for example, the different degrees of wear of the spool were produced by using laser processing equipment to produce grooves at different levels on the spool surface.

#### 3.2.3. Fault Data

**Figure 9:**
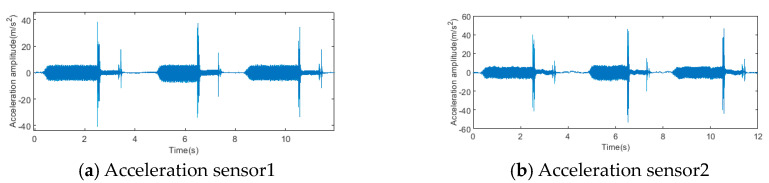

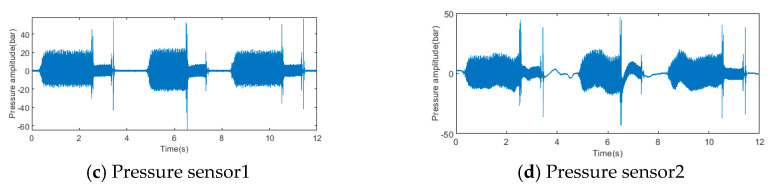
Time-series signal samples collected by four sensors.

Figure 9 displays the data obtained from two acceleration sensors and two pressure sensors, respectively, throughout a duration of 12 s. Fault samples were collected by means of window sliding. Each sample intercepted 1024 data points with a slip step size of 128. In each experiment, a single sensor can obtain 555 fault samples, so 6 different types of fault experiments are carried out, and 4 sensors can obtain a total of 4 × 555 × 6 samples.

## 4. Fault Diagnosis

**Figure 10:**
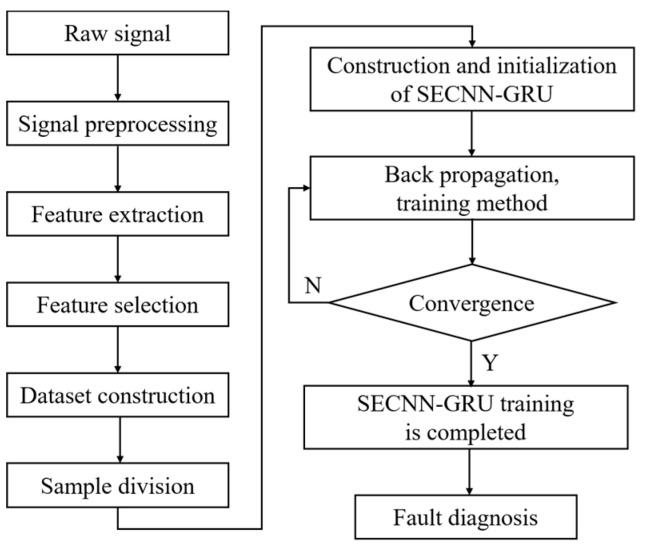
Fault diagnosis flow chart.

The fault diagnosis process based on SECNN-GRU is shown in Figure 10, which is mainly composed of data preprocessing, feature extraction, feature selection, dataset construction, sample division, diagnosis method construction, network training, and fault diagnosis.

### 4.1. Case1: Fault Diagnosis of the Hydraulic Multi-Way Valve

#### 4.1.1. Data Processing

The pressure experimental data of hydraulic multi-way valves under different fault states are obtained from Section 3. After preprocessing, 415 samples are obtained for each fault, totaling 1660 samples. 75% of the samples (1245 training samples) are used for classifier training, and 25% of the samples (415 testing samples) are used for classifier testing. To avoid specificity and contingency of the diagnosis results, ten tests are performed. Finally, the average test accuracy of ten tests is calculated.

#### 4.1.2. Feature Prioritization

**Figure 11:**
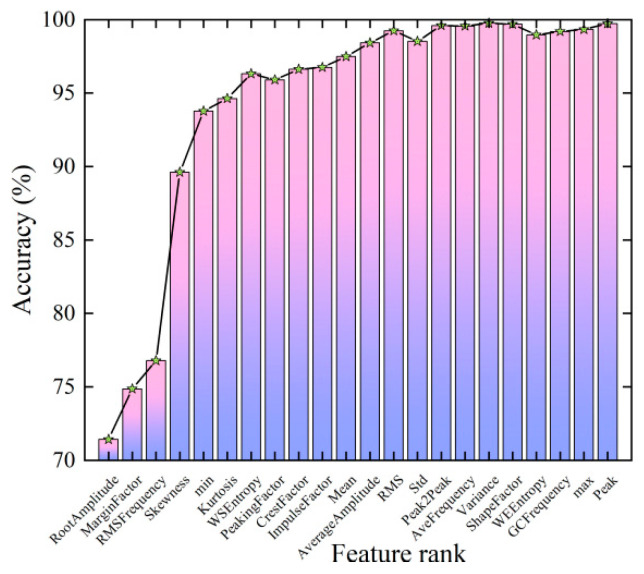
Feature ranking using mRMR algorithm.

After the pressure fault data is extracted in the time domain, frequency domain, and time-frequency domain, it is not guaranteed that the selected features are equally important in reflecting the health status of the hydraulic multi-way valve, so it is necessary to perform feature sorting and dimensionality reduction operations on the extracted features. As shown in Figure 11, CNN was used to conduct five classification accuracy experiments for the first several features after the optimization of mRMR features, and the average diagnostic accuracy was obtained. The horizontal coordinate represents the priority order of features after the sorting of mRMR features, and the vertical coordinate represents the diagnosis accuracy of the classifier. It can be seen that with the increase in the number of features, the diagnosis accuracy of the classifier first improves and then becomes stable, which further indicates that not all fault features are useful for diagnosis and that it is necessary to further optimize the features. The ability of fault feature characterization is improved, and the computational complexity is reduced.

#### 4.1.3. Results of Different Methods

The main parameter settings of the SECNN-GRU network are shown in Table 5. Firstly, the simulation signal is input into the SECNN network and the convolution operation is carried out. At the same time, the Squeeze-and-Excitation operation is performed. Then, the two are multiplied and assigned corresponding weights to obtain the weighted feature vector. The SECNN network is post-connected with Flatten, and data is input into the GRU network to further extract fault features. The final layer is the fully connected layer, which uses the Softmax function to divide the output into 4 categories. The hyperparameters are set as follows: the initial learning rate is 0.01, the learning rate drop factor is 0.1 after every 400 trainings, the batch size is 128, the maximum training number is 1000, and the loss function is the cross-entropy loss function.

**Figure 12:**
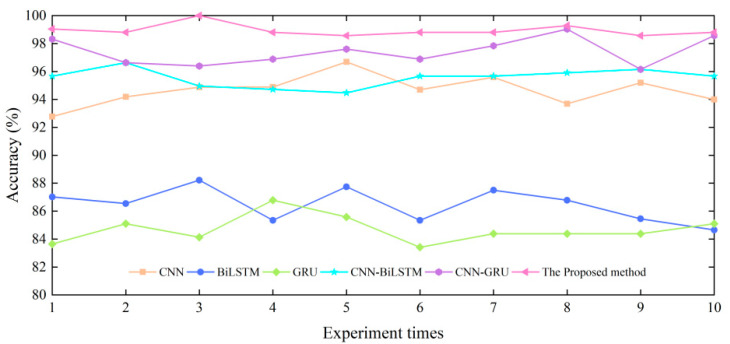
The accuracies of six different fault diagnosis methods under ten different sets.

Based on the above fault diagnosis methods, the accuracy and robustness are analyzed. Test samples are randomly selected from the total sample, and 10 consecutive tests are conducted on the selected test samples. The test results are shown in Figure 12. The method proposed in this paper has a high accuracy on the fault set of hydraulic multi-way valves. However, BiLSTM, GRU, CNN, etc. also have good classification performance, but the application occasions and conditions are different. Table 5 lists the main parameters of the BiLSTM, GRU, and CNN methods, respectively, and the setting of hyperparameters is the same as above. Methods with different structures show different results by using the same test set, and the method proposed in this paper is more accurate and robust than others. Therefore, the results show that the SECNN-GRU method proposed in this paper is more suitable for fault data diagnosis of hydraulic multi-way valves.

The average testing accuracy and standard deviation of various methods are shown in Table 6. The method proposed in this paper achieves an average testing accuracy of 98.94%, and the standard deviation is 0.40% on the test set, which is an improvement of 4.28%, 12.48%, 14.25%, 3.39%, and 1.51% over the CNN, BiLSTM, GRU, CNN-BiLSTM, and CNN-GRU network as inputs, respectively. It can be seen that the proposed method has significant advantages over the above methods in hydraulic multi-way valve fault diagnosis.

#### 4.1.4. Confusion Matrix

**Figure 13:**
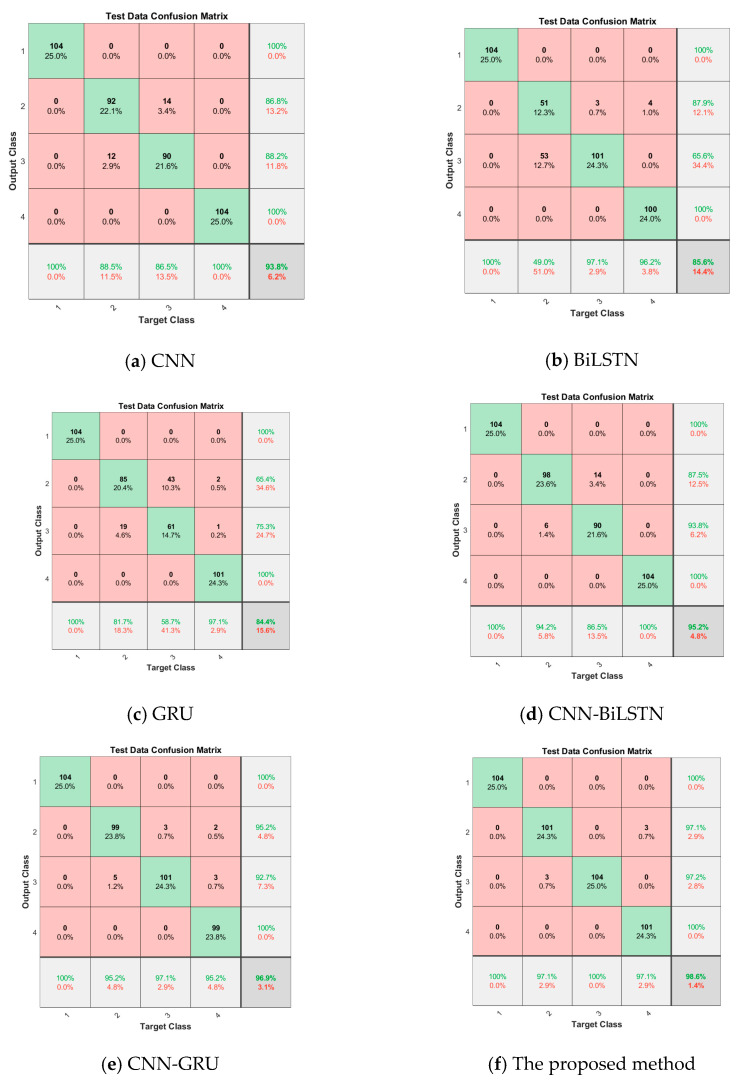
Testing samples confusion matrix for various methods.

Further analysis of the confusion matrix of this method is shown in Figure 13. The vertical axis represents the actual label of the sample, and the horizontal axis represents the predicted label of the sample. Labels 1, 2, 3, and 4 indicate normal, cavitation, severe, and moderate spring fault states, respectively. The generalization ability of the method is evaluated preliminarily by the confusion matrix. As can be seen from Figure 13f, the prediction label obtained by the fault diagnosis method proposed in this paper is more consistent with the actual label, indicating that the method has good fault recognition and classification ability.

#### 4.1.5. Influence of the Anti-Noise Performance

In this section, the anti-noise performance of the hydraulic multi-way valve dataset of the proposed method is discussed. Gaussian white noise with SNR=−3 dB∼3 dB is added to the simulation data in the training dataset samples, which is used to verify the generalization ability of the proposed method and the stability and reliability of fault diagnosis results. The formula for calculating the signal-to-noise ratio is shown in Equation (17).

(17) $$SNR=10log\frac{P_{signal}}{P_{noise}}$$

**Figure 14:**
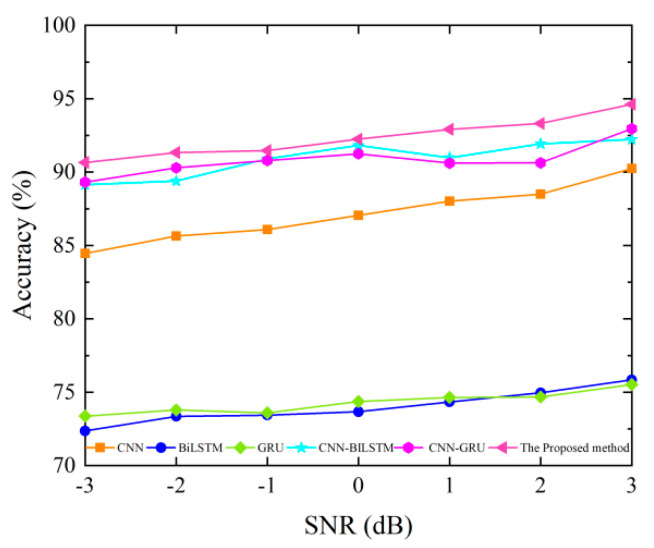
Classification accuracy of different methods under various SNRs on a hydraulic multi-way valve dataset.

As mentioned above, the CNN, BiLSTM, GRU, CNN-BiLSTM, and CNN-GRU are compared with the proposed method to verify the anti-noise performance. The noise-added test set is input into the trained diagnosis method, and the fault diagnosis accuracy obtained by different methods is shown in Figure 14.

As can be seen from the analysis in Figure 14, the proposed method has better anti-noise performance, and the generalization effect is significantly better than other methods in the comparison. When the white noise SNR is −3 dB, the diagnostic accuracy of the proposed method can reach more than 90%, while the diagnostic accuracy of CNN, BiLSTM, GRU, CNN-BiLSTM, and CNN-GRU methods are only 84.46%, 72.36%, 73.37%, 89.13%, and 89.31%, respectively. Therefore, it further proves that the proposed method has better fault diagnosis ability.

### 4.2. Case2: Hydraulic Directional Valve Fault Diagnosis Experiment

#### 4.2.1. Results of Different Methods

**Figure 15:**
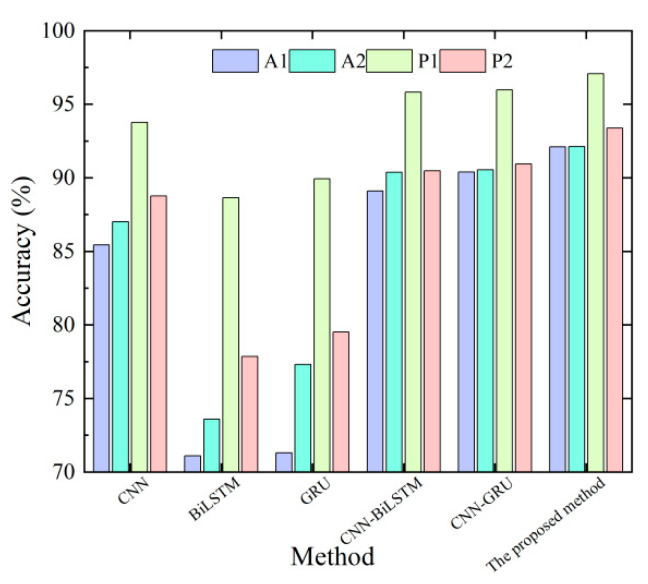
Average diagnostic accuracy of hydraulic direction valve under different methods.

For the fault data collected by the four sensors (acceleration sensors A1 and A2, pressure sensors P1 and P2), the same data processing and feature selection methods as in case 1 are adopted to obtain the fault features after the feature selection. As shown in Figure 15, after five experiments, the fault diagnosis accuracy results of different methods (parameter settings are the same as in case 1) are obtained. It can be seen that the results obtained by the proposed method are better than those obtained by other methods, and the data collected by the pressure sensor is more sensitive to faults.

The average diagnostic accuracy and standard deviation of each method are shown in Table 7. The average test accuracy of the proposed method on the test set is 92.10%, 92.12%, 97.07%, and 93.37%, and the standard deviation is 0.60%, 0.73%, 0.70%, and 0.83%, respectively. Compared with CNN, BiLSTM, GRU, CNN-BiLSTM, and CNN-GRU networks, the diagnostic accuracy of the method is improved to varying degrees, the performance effect is more stable, and the generalization ability is stronger. It can be seen that the proposed method has obvious advantages in the fault diagnosis of common-direction valves.

#### 4.2.2. Confusion Matrix

**Figure 16:**
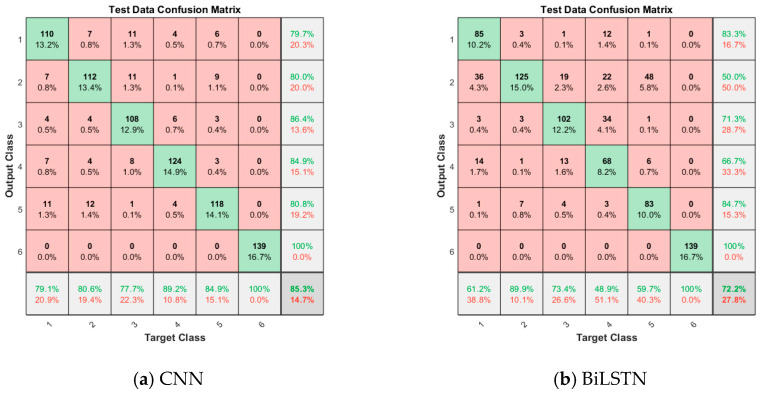

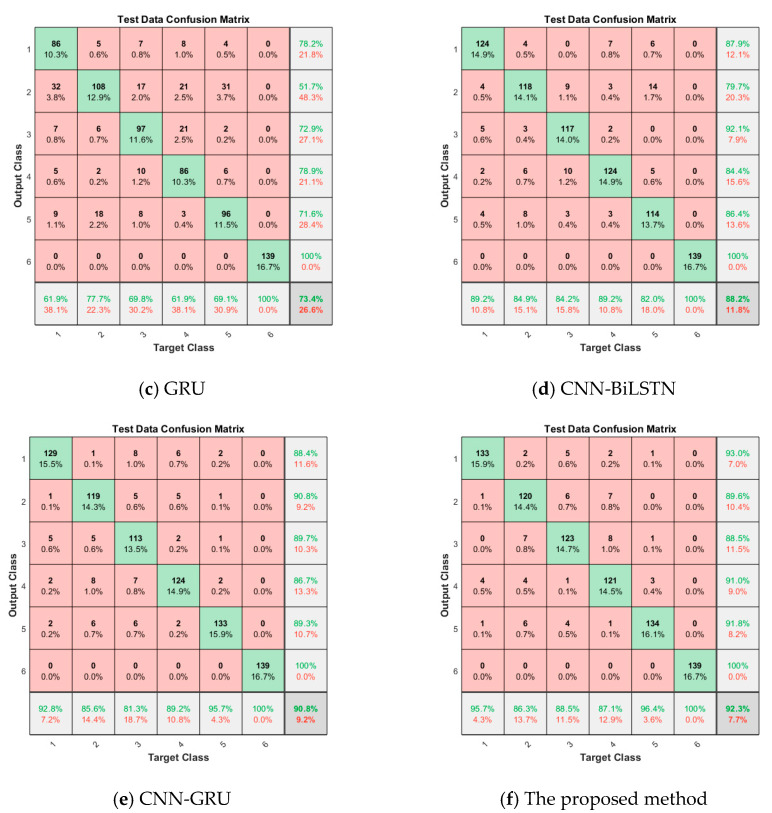
Testing samples confusion matrix for various methods.

Taking the fault data collected by acceleration sensor A1 as an example, the performance of the method is further analyzed through the confusion matrix. As shown in Figure 16, the vertical axis represents the actual label of the sample, while the horizontal axis represents the predicted label of the sample. Labels 1, 2, 3, 4, 5, and 6 represent normal, mild wear, moderate wear, severe wear, mild, and severe spring failure states, respectively. Overall, it can be seen from Figure 16 that the classification accuracy of the proposed method is improved to varying degrees compared with other methods, indicating that the method has good generalization ability. A detailed analysis of Figure 16f shows that a few samples in different categories are wrongly classified into other categories, which also reflects the defect of imperfect fault information contained by a single sensor to a certain extent. The fault diagnosis method of multi-sensor information fusion needs to be studied in the future.

## 5. Conclusions

This paper proposes a fault diagnosis method for a hydraulic multi-way valve based on an improved squeeze-excitation convolution neural network and gated recurrent unit method with mRMR feature selection. In this method, the optimal fault features are input into the intelligent diagnosis network to realize the fault diagnosis of hydraulic components. In order to solve the problem of the data source of the hydraulic multi-way valve, the simulation model is established and verified, and the fault data is obtained. The mRMR feature optimization algorithm optimizes the statistical features and obtains the optimal fault features. In order to improve the accuracy of fault diagnosis, the squeeze-and-excitation module is combined to assign different weights to the features.

In the test of hydraulic valve fault diagnosis, after several iterations, the average diagnostic accuracy of this method reaches 98.94% and 92.10% (A1 sensor as an example), respectively for the faults of hydraulic multi-way valves and hydraulic directional valves in different engineering fields. Compared with CNN, BiLSTM, GRU, CNN-BiLSTM, and CNN-GRU intelligent diagnosis algorithms, the standard deviation of the proposed method for hydraulic multi-way valve and hydraulic direction valve is 0.40% and 0.60% (A1 sensor as an example), respectively, which are smaller than other algorithms. The proposed method can effectively extract fault features and has good fault diagnosis performance, which verifies the effectiveness of the proposed method. Since incipient fault and missing-data working conditions are important research issues on fault diagnosis, we will conduct research on incipient fault and missing-data conditions in the future if the conditions are mature.

## Figures and Tables

**Table 1:** Feature types.

Time-Domain Feature				Frequency-Domain	Time-Frequency Domain
Max	Min	Mean value	Peak value	Gravity center frequency	Wavelet energy entropy
Peak-peak value	Average amplitude	Root amplitude	Variance	Root mean square frequency	Wavelet singular entropy
Standard deviation	Root mean square	Skewness	Kurtosis	Average frequency	
Waveform factor	Peak factor	Pulse factor	Margin factor		
Clearance factor					

**Table 2:** Parameter setting of AMESim simulation model of hydraulic multi-way valve.

Parameter	Value	Parameter	Value
Motor speed/(r/min)	1500	Spool displacement/(mm)	6
Pump displacement/(cc/rev)	25	Spring stiffness/(N/m)	0.01
Relief valve cracking pressure/(bar)	120	Preload/(N)	10
Spool diameter/(mm)	12	Density/(kg/$m^{3}$)	850
Rod diameter/(mm)	8	Temperature/(°C)	40
Spool mass/(kg)	0.15	Bulk modulus/(MPa)	1700
Radial clearance/(mm)	0.001	Flow coefficient	0.7

**Table 3:** Fault models of hydraulic multi-way valves.

Class	Fault Model	Fault Phenomenon	Fault Reason
1	Normal	None	None
2	Cavitation	Noise, Vibration, Efficiency reduction	Bubbles in the oil
3	Moderate failure of valve spring	Pressure regulation, pressure retention, and response function out of control	Fatigue, heat, and degradation
4	Seriously failure of valve spring		

**Table 4:** Fault models of hydraulic directional valve.

Class	Fault Model	Fault Phenomenon	Fault Reason
1	Normal	None	None
2	Light wear (0.015–0.035 mm)	Leakage	Oil particle pollution
3	Moderate wear (0.035–0.060 mm)		
4	Severe wear (>0.060 mm)		
5	Mild failure of return spring	Pressure regulation, pressure retention, and response function out of control	Fatigue, heat, and degradation
6	Severe failure of return spring		

**Table 5:** Main parameters of network structure.

CNN	BiLSTM	GRU	CNN-BiLSTM	CNN-GRU	SECNN-GRU
Conv_1, 8, 3 × 1, 1	Unit, 10	Unit, 10	Conv_1, 8, 3 × 1, 1	Conv_1, 8, 3 × 1, 1	Conv_1, 16, 3 × 1, 1
Maxpool, 2 × 1, 2	Fc, 4	Fc, 4	Maxpool, 2 × 1, 2	Maxpool, 2 × 1, 2	Maxpool, 2 × 1, 2
Conv_2, 16, 3 × 1, 1			Conv_2, 16, 3 × 1, 1	Conv_2, 16, 3 × 1, 1	Conv_2, 64, 3 × 1, 1
Maxpool, 2 × 1, 2			Maxpool, 2 × 1, 2	Maxpool, 2 × 1, 2	Maxpool, 2 × 1, 2
Conv_3, 32, 3 × 1, 1			Conv_3, 32, 3 × 1, 1	Conv_3, 32, 3 × 1, 1	Global average pool
Maxpool, 2 × 1, 2			Maxpool, 2 × 1, 2	Maxpool, 2 × 1, 2	Fc, 16
Conv_4, 32, 3 × 1, 1			Conv_4, 32, 3 × 1, 1	Conv_4, 32, 3 × 1, 1	Fc, 64
Maxpool, 2 × 1, 2			Maxpool, 2 × 1, 2	Maxpool, 2 × 1, 2	Flatten
Fc, 4			Unit, 10	Unit, 10	Unit, 10
			Fc, 4	Fc, 4	Fc, 4

Note: “Conv_a, b, c × d, e”, where a represents the name of the 1D convolution layer, b represents the number of convolution kernels, c × d represents the size of the convolution kernel, and e represents the parameter stride; “Maxpool, f × g, h” represents the 1D pooling layer, where f × g represents the pooling size and h represents the parameter stride; “Fc, m” represents the parameter of the full connection layer; “Unit, n” represents the number of internal units in the recurrent network.

**Table 6:** Average testing accuracy for various methods.

Methods	Average Accuracy(%)	Standard Deviation(%)
CNN	94.66	1.03
BiLSTM	86.46	1.14
GRU	84.69	0.94
CNN-BiLSTM	95.55	0.63
CNN-GRU	97.43	0.94
**The proposed method**	**98.94**	**0.40**

**Table 7:** Average diagnostic accuracy and standard deviation of hydraulic direction valves under different methods.

Methods	Average Accuracy(%)				Standard Deviation(%)			
	A1	A2	P1	P2	A1	A2	P1	P2
CNN	85.43	87.02	93.76	88.77	0.31	0.68	0.71	0.85
BiLSTM	71.10	73.59	88.65	77.86	0.33	0.89	0.62	0.72
GRU	71.30	77.31	89.94	79.52	0.46	0.66	0.74	0.92
CNN-BiLSTM	89.10	90.38	95.83	90.47	0.34	0.73	0.80	0.79
CNN-GRU	90.39	90.54	95.98	90.93	1.07	0.51	0.59	0.32
**The proposed method**	**92.10**	**92.12**	**97.07**	**93.37**	**0.60**	**0.73**	**0.70**	**0.83**

## Data Availability

Not applicable.
